# The development of a parental attachment recognition scale for mothers nurturing preschool children

**DOI:** 10.1111/phn.12697

**Published:** 2019-12-23

**Authors:** Yoko Tanaka

**Affiliations:** ^1^ Department of Nursing Faculty of Health Sciences Kio University Nara Japan; ^2^ Department of Home Health Care Nursing Graduate School of Nursing Osaka City University Osaka Japan

**Keywords:** attachment experience, maternal and child health, public health nurses, scale development

## Abstract

**Background:**

Attachment theory shows that childhood experiences influence the parenting style that the child shows later, as a parent. Nevertheless, at present there are no instruments to efficiently quantify the emotions associated with parents’ early attachment.

**Objective:**

This study aims to develop a cognitive scale that measures both the positive and negative emotions reflecting the early attachment experience of Japanese mothers now nurturing their own preschool‐age children.

**Design:**

A Parental Attachment Recognition Scale is developed in three phases that include concept analysis, item pool development and validity analysis, and investigation of the scale's statistical features, factor structure, validity, and reliability.

**Measurements and Sample:**

The scale development is based on a self‐administered questionnaire survey, answered by 639 mothers.

**Results:**

The scale consists of 27 items, grouped by factor analysis into three subordinate scales, named: (1) Parent–child contact; (2) Emotional bond; and (3) Parental impressions.

**Conclusions:**

The study proposes a new scale to measure the recognition of early childhood attachment experiences among mothers raising their own preschool children. The instrument has a considerable degree of validity and reliability. The scale is expected to be useful in helping public health nurses assess mothers in need of childrearing support.

## 1 BACKGROUND

In the “Healthy Parents and Children 21” report, published by the Ministry of Health, Labour, and Welfare of Japan (2014), the prevention of child abuse, starting from pregnancy, is listed as a priority. This is reflected in the number of child abuse consultation cases, which has followed an increasing trend, reaching 159,850 in 2018 (Ministry of Health, Labour, & Welfare, 2019).

Mothers raising children become aware of past negative childhood experiences, which are thought to affect their own childrearing practices (Madden et al., 2015). Prevention of child maltreatment requires breaking the intergenerational transmission chain (Berlin, Appleyard, & Dodge, 2011;Widom, Czaja, & DuMont, 2015). Factors that may prevent negative childhood experiences from being further transmitted include the ability of parents to reflect on their past, the presence of other important positive experiences during parents’ childhood, receiving social support, and a high educational background of parents (Cassidy & Shaver, 2016;Narayan, Rivera, Bernstein, Harris, & Lieberman, 2018).

Public health nurses should be able to recognize improper parental upbringing, and its impact on the mental health of children. To facilitate support, nurses should understand parents’ growth history and childhood experiences and encourage parents to reflect upon their past, while building a trusting relationship with the health care provider (Beebe, Lachmann, Markese, & Bahrick, 2012;Coffman, Levitt, & Guacci‐Franco, 1995;Nystrom & Ohrling, 2004).

Parent–child attachments—understood as long‐lasting emotional bonds—are crucial for healthy child development (Bowlby, 1973) and may impact the parenting style that the child shows later, as a parent (for example, Biringen, 1990). In the field of developmental psychology, the Adult Attachment Interview (Behrens, Hesse, & Main, 2007;George, Kaplan, & Main, 1984), which evaluates attachment patterns based on the respondent's childhood experience, the Parental Bonding Instrument (PBI) (Ogawa, 1991;Parker, Tupling, & Brown, 1979), which measures parental attitudes to upbringing, and the Inventory of Parent Attachment (IPA) (Armsden & Greenberg, 1987), which evaluates adolescents’ attachment to their parents, are widely used assessment tools. However, there are no studies on how to specifically understand the way parents perceive child‐care experiences based on their attachment experiences during childhood and there is no scale that quantifies these subjective early experiences.

### 1.1 Study purpose

The Parental Attachment Recognition Scale (PARS) developed in this study aims to quantify both the positive and negative emotions reflecting mothers’ early attachment experience. The specific content of the emotional connection with parents, from an attachment perspective, is explored using a cross‐sectional survey. Public health nurses may use this scale to assess mothers’ emotional background and support those concerned about their interaction with their children, as well as mothers with inappropriate upbringing practices. The scale may improve the ability of public health nurses to prevent child maltreatment.

## 2 METHODS

### 2.1 Analytic strategy

**Figure 1:**
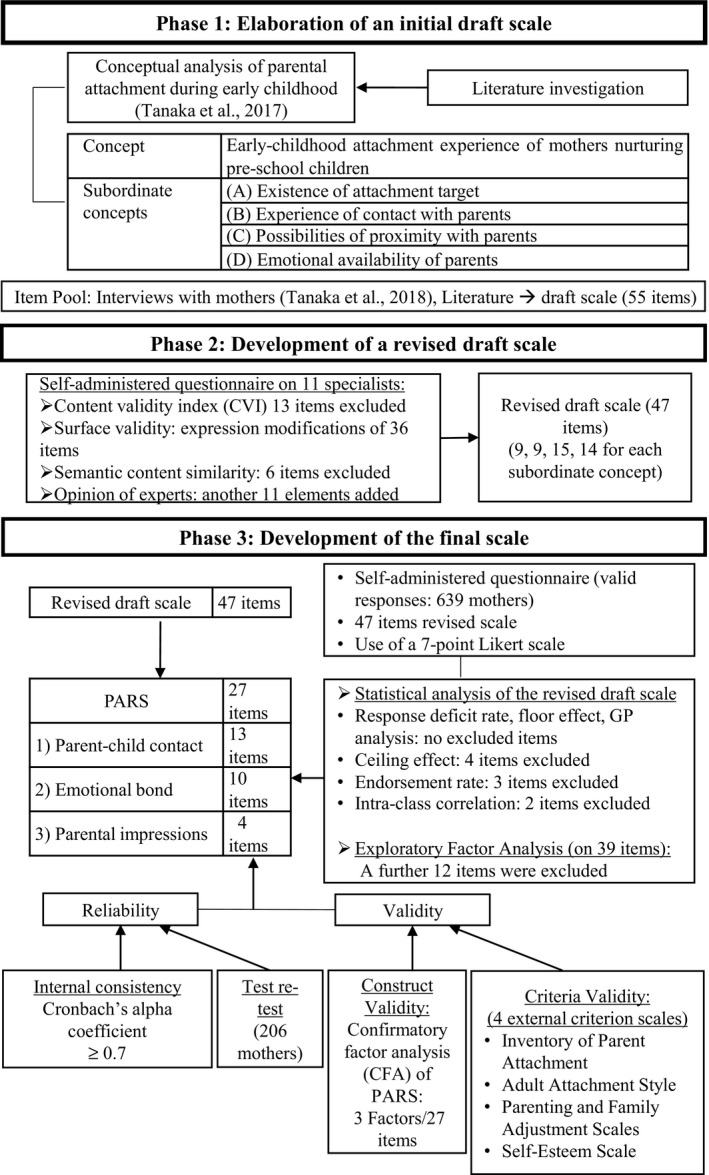
Overview of the three investigation phases that led to the development of PARS. PARS, Parental Attachment Recognition Scale

The development of PARS (Figure 1) went through the following three phases: (1) Elaboration of an initial draft scale, based on concept analysis and item pool development (Phase 1); (2) Development of a revised draft scale, based on the investigation of the content and surface validity of the draft scale (Phase 2); (3) Development of the final scale, based on the investigation of its statistical features, factor structure, reliability and validity (Phase 3—main phase). Phase 1 is only briefly described as it has been the focus of previous publications (Tanaka, Ueno, & Okawa, 2017, 2018).

### 2.2 Design and sample

#### 2.2.1 Elaboration of an initial draft scale (phase 1)

The constituent factors for the scale's elaboration were the following subordinate concepts of parental attachment during early childhood: (A) Existence of Attachment Target—relationship of the mother with her parents (i.e., target) in her childhood environment; (B) Experience of Contact with Parents—the mother's recognition of upbringing experiences; (C) Possibilities of Proximity with Parents—the mother's perception of the response received when seeking close interaction with her attachment figures; and (D) Emotional Availability of Parents—the mother's perception of the emotional involvement with her caregiver, obtained by concept analysis (Tanaka, Ueno, & Okawa, 2017), from a comprehensive investigation of relevant literature. All four subconcepts can be understood in the framework of the internal working models of Bowlby (1969), according to which early childhood experiences shape adult relationships.

The derivation of candidate scale items is based on the four subordinate concepts. The pool items were identified from semi‐structured interviews with mothers raising preschool children (25 items; Tanaka, Ueno, & Okawa, 2018) and knowledge from prior relevant literature (40 items; Tanaka et al., 2017). From the total of 65 items, 55 items were selected after carefully considering item compatibility and similarity.

#### 2.2.2 Development of a revised draft scale (phase 2)

A self‐administered questionnaire was completed by 11 specialists, with expertise in providing support with parent–child relationships, from January to March 2015. Respondents included three researchers specializing in maternal and child health, four public health nurses with at least 5 years of practical experience in maternal and child health, two psychologists involved in providing support for parent–child relationships, and two nursery school teachers with at least 5 years of working experience.

The content validity was investigated by evaluating the relevance and suitability of the draft scale items in relation to the subordinate concepts according to four grades (1 = “No relation”; 2 = “Relation cannot be judged without modification” or “There is a possibility of a lack of association even after modification”; 3 = “Related, but some minor modifications are required”; 4 = “Highly related”). Items with a score of less than 0.8 on the Content Validity Index (I‐CVI; Lynn, 1986) were excluded. The face validity was investigated by assessing expert opinions on the shortcomings, excesses, and expression of items.

#### 2.2.3 Development of the final scale (phase 3—main phase)

After selecting 13 study sites in Japan on the basis of expediency, a self‐administered questionnaire using the revised draft was conducted with a total of 1,119 mothers of preschool children (914 mothers with children attending nursery schools and 205 mothers participating in childrearing support initiatives). Respondents were requested to insert the completed survey forms in envelopes and drop them in collection boxes installed at nurseries. A researcher later collected the forms from the boxes. The survey was conducted between July and December 2015.

Retesting was conducted with 401 mothers at six sites by administering a second questionnaire 3 weeks after the first. To verify that the same respondents answered both the first and second questionnaires, anonymous ID numbers were used during the survey.

The following two sections describe in detail the measurements (questionnaire content) and data analyses performed to develop the final scale.

### 2.3 Measurements

The questionnaire contained questions on personal attributes of the mothers: age, academic background and birth order of the respondent among siblings, as well as the revised draft scale, and four external criterion scales described below. For the revised draft scale, each item is scored on a seven‐point Likert scale: 1 = Never true, 2 = Not true, 3 = Infrequently true, 4 = Neither true or false, 5 = Sometimes true, 6 = Usually true, 7 = Always true. The higher the score, the stronger is the recognition by mothers of their attachment experience during early childhood.

The IPA scale (Armsden & Greenberg, 1987), Japanese version (Fujii, 1994), is a self‐report instrument for use with adolescents. The subordinate scales are "Communication", "Alienation", and “Trust”. The Adult Attachment Style (AAS) scale (Takuma & Toda, 1988) measures the interpersonal relationships of mothers. The subordinate scales are “Secure”, “Avoidant”, and "Anxious". The Parenting and Family Adjustment Scales (PAFAS) (Sanders, Morawska, Haslam, Filus, & Fletcher, 2014), Japanese version (Fujioka, Tanaka, & Wakimizu, 2016) is an outcome measure for assessing changes in parenting practices and parental adjustment. PAFAS has two scales: "Parenting"; and "Family Adjustment". The “Parenting” scale includes four factors: “Parental Consistency", "Coercive Parenting"; "Positive Encouragement"; and "Parent–child Relationship", while the “Family Adjustment” scale has three factors: "Parental Adjustment"; "Family Relationships"; and "Parental Teamwork". The Self‐Esteem Scale (SES) (Rosenberg, 1965; Japanese version: Mimura & Griffiths, 2007; Uchida & Ueno, 2010) measures both positive and negative feelings about the self.

### 2.4 Data analysis

#### 2.4.1 Statistical features

Items with an answer deficit rate of 5% or more are considered problematic and should be excluded (Streiner & Norman, 2008). A ceiling effect occurs if the sum of the average and standard deviation for an item equals seven (upper limit of the scale) or more, while a floor effect is observed if the average minus the standard deviation equals one or less. The endorsement rate represents the proportion of those who answered, "Always true" or "Usually true", in the case of positive items, and "Never true" or “Not true”, in the case of negative (reverse) items. Endorsement rates of 20%–80% are considered appropriate (Streiner & Norman, 2008). A *t* test was conducted on the difference between the upper 75‐percentile and lower 25‐percentile rank groups of answers for each item (Good–Poor [GP] analysis). The Item‐Remainder (IR) correlation examines the correlation between each item score and the total score of the remaining items. Items showing very low correlation (<0.1) are excluded since they are considered inconsistent (i.e., not correlated) with the content being measured on the scale (Churchill, 1979).

#### 2.4.2 Factor analysis

The Exploratory Factor Analysis (EFA) was done on the items selected on the basis of the results of the statistical analysis outlined above. EFA was performed using maximum likelihood factor analysis to determine the number of factors (underlying latent variables), followed by Promax rotation to determine the final factor loadings. Items were excluded after comprehensively judging their factor loading and commonality. Groups of items with factor loadings of 0.4 or more and commonality of 0.3 or more were set to define the subscales (Hair, Black, Babin, Anderson, & Tatham, 2010). Processing by excluding “weak” items was carried out and repeated analyzes were conducted until obtaining the most reasonable factor structure.

#### 2.4.3 Scale reliability: internal consistency and stability

The internal consistency was assessed using Cronbach's α coefficient calculated for each subscale, as well as for the whole scale. An α coefficient of 0.7 or more is considered reasonable (Nunnally, 1978). The stability of the scale at repeated measurements was assessed using the test‐retest method and calculating the Intra‐class Correlation Coefficient (ICC). An ICC of 0.7 or more is recommended (Streiner & Norman, 2008).

#### 2.4.4 Scale validity: criterion‐related validity and construct concept validity

The validity of the scale items extracted by factor analysis was assessed in terms of criterion‐related validity and construct concept validity. The concurrent validity (one type of criterion‐related validity) was assessed based on the degree of correlation (Dijkstra, Buist, & Dassen, 1998) between external criterion scales and our subordinate scales. The construct concept validity was addressed based on Confirmatory Factor Analysis (CFA), by assessing whether the factor structure model obtained as a result of EFA is compatible with the data. The fitness of the model was evaluated by the Comparative Fit Index (CFI) and Root Mean Square Error of Approximation (RMSEA). The statistical analyses were performed using the IBM SPSS Statistics Ver. 23 and Amos Ver. 18 software packages.

### 2.5 Ethical considerations

The study was conducted with the approval of the Ethical Research Review Board at Osaka Prefecture University, Graduate School of Nursing (approval nos. 25–65, 26–54 and 27–22).

## 3 RESULTS

Subsection 3.1 concerns results obtained during Phase 2, while the following subsections present results of Phase 3 of the scale elaboration.

### 3.1 Revised scale items

Out of 55 items, a total of 19 (13 items with I‐CVI <0.8, and six of similar semantic content) were excluded. Eleven items were then added and revised based on expert opinions, and a revised draft scale consisting of 47 items was prepared (Table 1).

**Table 1:** List of the 47 items of the revised draft scale. Items 1–9, 10–18, 19–33, and 34–47 belong to subconcepts (A), (B), (C), and (D), respectively. Items in bold print are those in the final scale (27 items)

Item no.	Content	I‐CVI
1	My parents were doing their best, with a positive attitude	0.91
2	**My parents found childrearing tiring (R)**	0.82
3	**My parents always had smiling faces**	New item^a^
4	**My parents were gentle**	New item
5	My parents acted well	New item
6	**My parents were always irritated (R)**	New item
7	My parents enjoyed housework	New item
8	My parents were busy with work (R)	New item
9	**My parents doted on me**	New item
10	**My parents held me when I asked them to**	0.91
11	**My parents took me to my favorite places**	0.91
12	My parents scolded me severely for mischief (R)	1.00
13	My parents encouraged me to do my best even with things that were unpleasant to me (R)	0.82
14	My parents sometimes hit me when I was doing something dangerous	1.00
15	My parents made me rephrase when my words were not well chosen	New item
16	**My parents read picture books to me when I asked them to**	New item
17	My parents persisted in giving me instructions, even when I was unwilling to listen (R)	New item
18	**My parents made handmade items I liked**	New item
19	My parents spoilt me	1.00
20	**My parents listened carefully when I was talking to them**	1.00
21	My parents accompanied me when I was sick	0.91
22	**My parents let me play freely when I wanted**	0.91
23	**My parents listened to me without hesitation when I was in trouble**	1.00
24	**My parents praised me when I was helping**	1.00
25	**My parents paid attention to what I was doing**	0.91
26	**My parents enjoyed playing with me or talking to me**	0.91
27	**My parents were smiling when I was around**	0.91
28	**My parents talked to me in a warm and affectionate voice when I was uneasy**	0.91
29	My parents helped out when I was in trouble	1.00
30	**My parents let me do things I was able to do**	1.00
31	My parents did not seem to enjoy it when we were together	0.91
32	My parents did not stay with me when I wanted them to be around	0.90
33	My parents were sometimes getting angry at me for the same thing	0.90
34	**My parents gave me the feeling of security that I was always loved**	1.00
35	**My parents understood my feelings**	1.00
36	**My parents were always kind to me**	1.00
37	My parents raised me carefully	1.00
38	**My parents always appreciated my thoughts and opinions**	1.00
39	**My parents watched over my conduct**	1.00
40	**My parents accepted me as I was**	0.91
41	My parents were stricter with me than with my siblings (R)	1.00
42	My parents were overprotective of me (R)	1.00
43	My parents allowed me to act at own pace (R)	0.82
44	**My parents were cold to me (R)**	1.00
45	**My parents did not believe me (R)**	1.00
46	**My parents did not notice when I was feeling uncomfortable (R)**	1.00
47	My parents understood my needs without me having to tell them	1.00

Note

(1) (R) denotes a reverse item. (2) Each item was scored on a 7‐point Likert scale, as detailed in the text.

^a^ “New item”: newly added items (11) based on expert opinion.

### 3.2 Self‐administered questionnaire respondents

From a total of 1,119 mothers, responses and informed consent were obtained from 767 (68.5% response rate). There were 639 mothers who provided valid responses (57.1% effective response rate). The average age of mothers was 34.5(±5) years old (range 18–53). Among the 639 mothers, 415 (64.9%) were raised in nuclear families, 198 (31.0%) in extended families, 18 (2.8%) in single mother families, five (0.8%) in single father families, two (0.3%) by grandparents and one (0.2%) in an orphanage. There were 73.7% of the mothers working at the time of the interview. Of the 231 mothers who consented to the retest (57.6% response rate), 206 (51.4% effective response rate) provided valid answers.

### 3.3 Statistical examination of items

The answer deficit rate for the items was 0%–1.95% (smaller than the 5% threshold), showing that the scale content was easy for the respondents to understand. A ceiling effect was recognized for items 1, 21, 29, 37, reflecting affectionate, positive attitudes of parents; it can be inferred that the mothers were well aware of their parents’ affection throughout childrearing. Items 12, 15, 43 had endorsement rates of less than 20%, likely indicating behavior that most people do not engage in (Streiner & Norman, 2008). Based on the GP analysis, significant differences between the lower and higher rank groups were found for all items, indicating a relatively high discriminatory power. Items 12, 42 had low IR correlation; they evaluate parents’ attitudes to childrearing, as perceived by their children, so it is likely that the correlation was low because the descriptions they contain did not correspond with the attachment experience of the subjects.

The eight items above, which did not satisfy the criteria of statistical analysis, were excluded. EFA was conducted on the remaining 39 items.

### 3.4 Exploratory factor analysis

Three factors were obtained considering the number of eigen values of the correlation matrix greater than 1.0 and scree plot analysis. A Maximum Likelihood Promax rotation was performed, and 9 items (5, 7, 8, 19, 31, 32, 33, 41 and 47) with a factor loading of less than 0.4 and three items (13, 14, and 17) with a commonality less than 0.3 were excluded, resulting in a 27 item‐scale, composed of three factors (i.e., subordinate scales), named: 1) Parent–child contact; 2) Emotional bond; and 3) Parental impressions (Table 2). The cumulative contribution rate was of 58.2%. The correlation coefficient between the three subscales was in the range of 0.69–0.82, showing a significant positive correlation.

**Table 2:** EFA results. The 27 final scale items and 3 factors are listed in English, as translated from the original Japanese version

Item		First factor	Second factor	Third factor	CO
Factor 1: Parent–child contact: α = 0.94, ICC = 0.8					
26	My parents enjoyed playing with me or talking to me	**0.840**	0.048	0.022	0.802
27	My parents were smiling when I was around	**0.720**	0.147	0.047	0.771
24	My parents praised me when I was helping	**0.688**	0.126	−0.067	0.553
16	My parents read picture books to me when I asked them to	**0.681**	−0.105	0.039	0.395
28	My parents talked to me in a warm and affectionate voice when I was uneasy	**0.661**	0.257	0.017	0.797
25	My parents paid attention to what I was doing	**0.657**	0.198	−0.003	0.797
10	My parents held me when I asked them to	**0.637**	0.025	0.116	0.557
18	My parents made handmade items I liked	**0.614**	−0.151	0.004	0.254
20	My parents listened carefully when I was talking to them	**0.601**	0.295	−0.027	0.696
11	My parents took me to my favourite places	**0.553**	0.055	0.020	0.375
9	My parents doted on me	**0.535**	−0.114	0.171	0.336
23	My parents listened to me without hesitation when I was in trouble	**0.520**	0.365	−0.040	0.657
34	My parents gave me the feeling of security that I was always loved	**0.490**	0.273	0.104	0.653
Factor 2: Emotional bond: α = 0.93, ICC = 0.9					
40	My parents accepted me as I was	−0.175	**1.004**	0.015	0.776
38	My parents always appreciated my thoughts and opinions	−0.170	**0.973**	0.076	0.803
39	My parents watched over my conduct	−0.075	**0.922**	0.020	0.771
45	My parents did not believe me (R)	0.017	**−0.667**	0.060	0.526
30	My parents let me do the things I was able to	0.180	**0.566**	−0.138	0.387
22	My parents let me play freely when I wanted to	0.207	**0.560**	−0.138	0.408
35	My parents understood my feelings	0.252	**0.515**	0.093	0.647
44	My parents were cold to me (R)	0.217	**−0.470**	0.112	0.553
46	My parents did not notice when I was feeling uncomfortable (R)	0.175	**−0.449**	0.109	0.467
36	My parents were always kind to me	0.125	**0.436**	0.307	0.634
Factor 3: Parental impressions: α = 0.83, ICC = 0.9					
4	My parents were gentle	−0.011	−0.022	**0.893**	0.776
6	My parents were always irritated (R)	−0.040	0.106	**−0.654**	0.494
3	My parents always had smiling faces	0.240	−0.048	**0.645**	0.637
2	My parents found childrearing tiring (R)	0.074	−0.011	**−0.531**	0.334
	Factor 1	—	0.823**	0.698**	
	Factor 2	0.823**	—	0.685**	
	Factor 3	0.698**	0.685**	—	

Note

(1) CO (third column of the table) stands for Commonality. (2) Maximum likelihood factor analysis with Promax rotation. (3) (R): Reversed item. (4) Pearson's coefficient of correlation **p < .01.

Abbreviations: EFA, Exploratory Factor Analysis; ICC, Intra‐class Correlation Coefficient.

Bold indicates factor loadings equal or larger than 0.4 for ordinary items and equal or less than ‐.4 for reversed items.

The items excluded by EFA reflect general content that is not consistent with the overall structure and content of the scale; for example, they imply an evaluation by the respondent of her parents’ attitudes to childrearing or relate to feeling unfulfilled during childhood, which the respondent may have had difficulties in accommodating.

### 3.5 Reliability investigation

The Cronbach's α coefficients were 0.83, 0.94, 0.93, and 0.83, for the whole‐scale and the first, second, and third subscales, respectively. The ICC, measuring the consistency of repeatedly measured continuous data (i.e., test and re‐test in our case), was 0.8, 0.7, 0.8, and 0.8, for the whole scale, and the first, second, and third subscales, respectively.

### 3.6 Validity investigation

Criterion‐related validity assessment (Table 3) showed that our subordinate scales positively correlate with “Communication” and “Trust” of the IPA scale, with “Secure” on the AAS scale and with the SES. Moreover, significant negative correlations were observed with “Alienation” on the IPA scale, with “Anxious” and “Avoidant” on the AAS scale and the PAFAS.

**Table 3:** Criterion‐related validity results

	Factor 1	Factor 2	Factor 3
IPA Communication	0.700**	0.699**	0.513**
IPA Alienation	−0.568**	−0.680**	−0.579**
IPA Trust	0.667**	0.802**	0.566**
AAS Secure	0.351**	0.355**	0.320**
AAS Anxious	−0.302**	−0.372**	−0.352**
AAS Avoidant	−0.363**	−0.347**	−0.317**
PAFAS Parental consistency	−0.109**	−0.078	−0.088*
PAFAS Coercive Parental	−0.120**	−0.147**	−0.205**
PAFAS Positive Encouragement	−0.203**	−0.222**	−0.093*
PAFAS Parent–child Relationship	−0.338**	−0.328**	−0.219**
PAFAS Parental Adjustment	−0.288**	−0.337**	−0.269**
PAFAS Family Relationship	−0.259**	−0.299**	−0.269**
PAFAS Parental Teamwork	−0.197**	−0.217**	−0.182**
SES	0.310**	0.352**	0.281**

Note

Spearman's correlation coefficient **p < .001.

Abbreviations: AAS, Adult Attachment Style; IPA, Inventory of Parent Attachment; PAFAS, Parenting and Family Adjustment Scales; SES, Self‐Esteem Scale.

**Figure 2:**
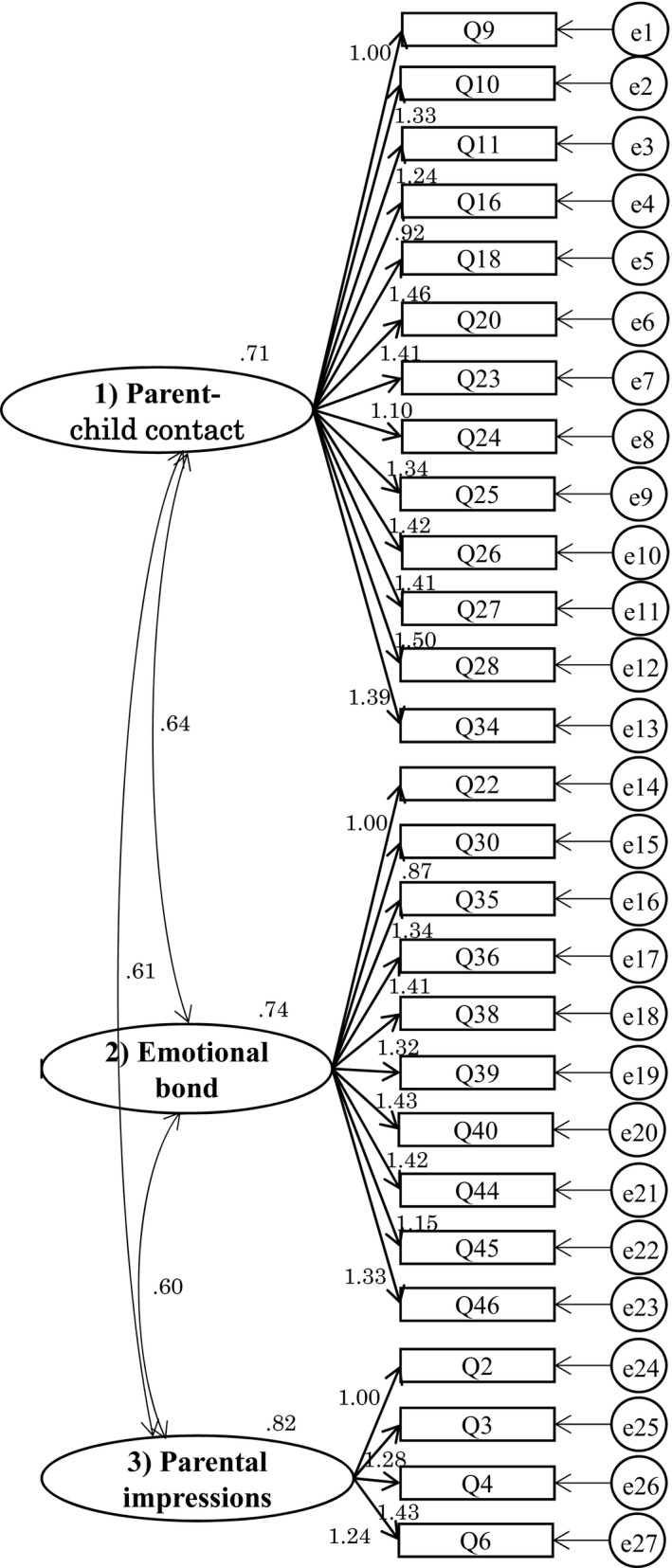
Path diagram (non‐standardized graphic output) for the CFA of PARS. There are 27 items (observed variables) that are grouped in three factors (latent variables). Numbers near straight arrows indicate factor loadings; those near curved arrows show the correlations between factors. CFA, Confirmatory Factor Analysis; PARS, Parental Attachment Recognition Scale.

A CFA model (Figure 2) for the 27 items and three subordinate scales was constructed by covariance structure analysis and it was examined how well it could explain the data. As a result, the goodness‐of‐fit indices were CFI = 0.90 and RMSEA = 0.08. All latent variables showed significance at the 5% level.

## 4 DISCUSSION

### 4.1 Scale factor interpretation

As noted in Section 2.2, the initial draft scale was developed on the basis of four subordinate concepts; however, it has become a three‐factor structure. This can be explained by the fact that the subconcepts (B) Experience of contact with parents and (C) Possibilities of proximity with parents can be interpreted as (1) Parent–child contact. The subordinate concept (D) Emotional availability of parents is regarded as (2) Emotional bond, and the subordinate concept (A) Existence of attachment targets is viewed as (3) Parental impressions. Note that most of the items in Factor (1) are part of subordinate concepts (B) and (C), items in (2) are part of (D) and items in (3) are part of (A). There were only four exceptions.

The scale items of Factor 1 concern close parent–child interactions, important for the attachment experience. Thus, items 10, 24, 27, and 28 describe the mother's feelings when her parents understood her mood and expressions. Items 11, 16, 18, 20, 23, and 25 reflect parents’ receptivity to children's wishes. Items 9, 26, and 34 reflect the child's confidence of being cared for. Factor 1 was named "Parent–child contact", suggesting a warm atmosphere of care‐giving and a feeling of security and affection. These items are considered to have an important influence on mothers' parenting style (Biringen, 1990).

Items 30, 38, 39, and 46 of Factor 2 reflect parents’ attitudes toward the child's initiatives or unpleasant feelings. Items 22, 35, 36, and 40 express an understanding behavior by the parents, while nurturing the child's trust. Items 44 and 45 reflect mothers’ unfulfilled wishes. Factor 2 has been named “Emotional bond”. The emotional bonds (Bowlby, 1973) between parents and children establish an atmosphere of mutual trust that helps children become secure and autonomous.

Items 3 and 4, of Factor 3, express positive parental impressions, while items 2 and 6 reflect negative ones. Factor 3 has therefore been named “Parental impressions”. Mothers’ subjective parental impressions may provide valuable complementary information on the quality of attachment during their childhood.

### 4.2 Scale reliability and validity

The Cronbach's α and ICC coefficients confirmed the internal consistency and stability of the scale. These results indicate a certain degree of reliability of the scale.

Remarkably, there was a strong positive correlation between our subscales and the subscales "Communication" and "Trust" of IPA. A negative correlation was found between our subscales and PAFAS, IPA’s “Alienation” and AAS’s “Anxious” and “Avoidant” subscales. These results confirm that a higher score on PARS subscales reflects a stronger recognition of the attachment experience. IPA was essentially developed to evaluate adolescents’ perceptions of their parental attachment. The relatively strong correlations with IPA indicate that the current attachment of mothers to their parents is deeply related to their attachment experience during early childhood.

The CFI value is close to 0.9, which indicates a good fit of the model (Bentler, 1990). The RMSEA <0.08 can be interpreted as a fair fit (Fabrigar, Wegener, MacCallum, & Strahan, 1999). It can therefore be concluded that the 3‐subscale model is successfully confirmed by CFA.

### 4.3 Novel developments and possible applications in nursing practice

Previous research has demonstrated a close relationship between attachments during childhood and parenting experience during adulthood (for example, Biringen, 1990). In this context, PARS reveals the subjective recognition of mothers of their childhood attachment and offers insight into their current parenting practice.

Since PARS is constructed based on the four attachment subconcepts described above, it reflects the emotional content of attachment experiences during childhood, rather than attitudes toward parenting or recollections, as in previous work (for example, PBI, Parker et al., 1979). While developed based on Japanese mothers’ experiences, the generality of the scale's questions would make it relatively easy to extend to different cultural and social contexts.

Parental Attachment Recognition Scale may serve as an effective, relatively fast to administer but robust questionnaire‐based assessment tool for problematic mother–child attachments. The seven‐level self‐assessment questionnaire takes about 5 min to implement, thus making it suitable for use in nursing practice, during home visits and regular infant check‐ups.

The scale is also expected to have an impact on the work of professionals specializing in the development and support of parent–child relationships (Zeanah, Berlin, & Boris, 2011). Reinforcing the recognition of positive past attachment experiences (Lieberman, Padrón, Van Horn, & Harris, 2005;Narayan et al., 2018) and reflecting on the negative ones (Madden et al., 2015) would help mothers’ current childrearing endeavors.

### 4.4 Limitations of research and future issues

Considering the possible utilization of attachment assessment tools during pregnancy, utilization of the scale in maternal and child health care activities should be broadened. Furthermore, verification of the scale's applicability is needed using more diverse samples that reflect ethnic and cultural differences.

It is also desirable to assess mothers’ individual backgrounds, which would help clarify the factors affecting their perceptions of childrearing and enhance the appropriate support methods. Since there might be some resistance from mothers with negative childhood experiences to answer the questionnaire, it may be necessary first to build up a trusting relationship with the respondent, before applying the assessment tool and providing support.

Besides obtaining information on the relationship with parents in early childhood, there is a need to gather information about the possible existence of attachment with adults other than parents, about changes in the relationship with parents, and current childrearing practices.

## 5 CONCLUSIONS

The present study proposes a PARS, based on a thorough investigation that took place in three phases. The scale is expected to prove useful for public health nurses in community settings, when supporting mothers with concerns about their parental involvement. The scale will enable nurses to reflect, together with the mothers, on the mothers' own attachment experiences during childhood, deepen their understanding of the mothers, and provide support based on the mothers’ own experiences and emotions.

## ETHICAL STATEMENT

The study was conducted with the approval of the Ethical Research Review Board at Osaka Prefecture University, Graduate School of Nursing (approval nos. 25–65, 26–54 and 27–22).
